# Postmastectomy irradiation in breast in breast cancer patients with T1-2 and 1-3 positive axillary lymph nodes: Is there a role for radiation therapy?

**DOI:** 10.1186/1748-717X-6-28

**Published:** 2011-03-30

**Authors:** Rusen Cosar, Cem Uzal, Fusun Tokatli, Bengu Denizli, Mert Saynak, Nesrin Turan, Sernaz Uzunoglu, Alaattin Ozen, Atakan Sezer, Kamuran Ibis, Burcu Uregen, Vuslat Yurut-Caloglu, Zafer Kocak

**Affiliations:** 1 Trakya University Hospital, Department of Radiation Oncology, Edirne, TURKEY; 2 Medicana Hospital, Department of Radiation Oncology, Istanbul, TURKEY; 3 Trakya University Hospital, Department of Biostatistics, Edirne, TURKEY; 4 Trakya University Hospital, Department of Internal Medicine, Division of Medical Oncology, Edirne, TURKEY; 5 Trakya University Hospital, Department of Surgery, Edirne, TURKEY

## Abstract

**Background:**

We aimed to evaluate retrospectively the correlation of loco-regional relapse (LRR) rate, distant metastasis (DM) rate, disease free survival (DFS) and overall survival (OS) in a group of breast cancer (BC) patients who are at intermediate risk for LRR (T1-2 tumor and 1-3 positive axillary nodes) treated with or without postmastectomy radiotherapy (PMRT) following modified radical mastectomy (MRM).

**Methods:**

Ninety patients, with T1-T2 tumor, and 1-3 positive nodes who had undergone MRM received adjuvant systemic therapy with (n = 66) or without (n = 24) PMRT. Patient-related characteristics (age, menopausal status, pathological stage/tumor size, tumor location, histology, estrogen/progesterone receptor status, histological grade, nuclear grade, extracapsular extension, lymphatic, vascular and perineural invasion and ratio of involved nodes/dissected nodes) and treatment-related factors (PMRT, chemotherapy and hormonal therapy) were evaluated in terms of LRR and DM rate. The 5-year Kaplan-Meier DFS and OS rates were analysed.

**Results:**

Differences between RT and no-RT groups were statistically significant for all comparisons in favor of RT group except OS: LRR rate (3%vs 17%, p = 0.038), DM rate (12% vs 42%, p = 0.004), 5 year DFS (82.4% vs 52.4%, p = 0.034), 5 year OS (90,2% vs 61,9%, p = 0.087). In multivariate analysis DM and lymphatic invasion were independent poor prognostic factors for OS.

**Conclusion:**

PMRT for T1-2, N1-3 positive BC patients has to be reconsidered according to the prognostic factors and the decision has to be made individually with the consideration of long-term morbidity and with the patient approval.

## Introduction

Modified radical mastectomy (MRM) is an important treatment for many breast cancer (BC) patients especially with diffuse local disease and generally it is accepted safe for local control in treatment of patients with T1-2 and 1-3 positive axillary lymph nodes. Although, the role of adjuvant systemic treatments is relatively clear because numerous randomized clinical trials have established that adjuvant chemotherapy and/or hormonal treatment prolongs the survival of patients in this group. The role of post-mastectomy radiotherapy (PMRT) is the most controversial issue for adjuvant breast cancer management [1].

In subgroup analysis of Denmark 82b-c trials (DBG) it is strongly indicated that the benefit of PMRT is equally pronounced in patients with 1-3 positive nodes as in patients with 4 and more positive nodes [2-4]. Decision for making PMRT according to the number of positive lymph node status is controversial due to discrepancies in reported baseline LRR risks [5]. The National Cancer Institute of Canada Clinical Trials Group MA25 study was designed to assign in patients with 1-3 positive nodes to receive either loco-regional RT or no-RT after MRM randomly. However, this study was closed because of lack of accrual. Another closed study (MA20), which was conducted by the same group, included patients who had undergone breast-conserving surgery with high-risk node positive, and node negative, were randomly allocated to receive standard only breast RT versus locoregional RT. Nevertheless, this study could not be an answer to our question because the allocated patients had undergone breast conserving surgery therefore, were subjected to receive planned breast RT. The particular question, which requires a precise answer by the radiation oncologists, is the rate of LRR in these 1-3 lymph node positive patients who never received RT following MRM. Additional two questions are also important: would disease free survival (DFS), even overall survival (OS) could be affected by PMRT. Unfortunately, the answer will wait to be clarified by randomized trials in coming years. Ongoing randomized SUPREMO study was designed to evaluate the results of chest wall irradiation in management of the patients underwent MRM with pT1N0M0 or pT2N0-1M0 disease. It may give us better information the role of PMRT in this patient group [6].

Radiation oncologists often confront patients with 1-3 positive lymph nodes following MRM in their routine clinical practice and which factors should be considered as prognostic risk factors when deciding whether a patient should receive RT to chest wall with or without peripheral lymphatics, is a hard to make decision. Based on DBG report, we decided to evaluate our patients retrospectively [2-4]. We aimed to evaluate the correlation of loco-regional relapse (LRR) rate, distant metastasis (DM) rate, DFS and OS in a group of BC patients at intermediate risk for LRR (T1-2 and 1-3 positive axillary nodes) treated with or without RT following MRM.

## Materials and methods

We evaluated 600 BC patients treated or had their follow-up at our clinic from July 1999 to December 2006. Ninety BC patients, who had undergone MRM and had T1-T2 and 1-3 positive axillary lymph nodes, all but one of whom received adjuvant systemic therapy with (n = 66) or without (n = 24) PMRT were analysed. Fifteen of 90 patients were previously treated in another clinic prior to July 1999 (first patient operated at January 1992) and had their follow-up at our clinic. All patients underwent MRM with clear surgical margins (>1 mm). Axillary lymph node staging was performed in all patients. Pathological staging was reviewed based on AJCC 2002. The date of evaluation was January 2009.

Patient-related characteristics (age, menopausal status, pathological stage/tumor size, tumor location, histology, estrogen/progesterone receptor status, histological grade, nuclear grade, extracapsular extension, lymphatic, vascular and perineural invasion, and ratio of involved nodes/dissected nodes), and treatment-related factors (PMRT, chemotherapy and hormonal therapy) were analyzed (Table 1).

**Table 1:** Clinical characteristics of patients undergoing modified radical mastectomy

Groups	Radiotherapy n = 66		No-Radiotherapy n = 24	
**Characteristic**	**n**	**(%)**	**n**	**(%)**

**Age, years**				
**Median**	51	48	50	50
**Range**	28-73		34-77	
**≤50**	32		12	

**Postmenopausal status**	37	56	13	54

**Lymph nodes examined**				
**Median**	12		10	
**Range**	(3-37)		(4-31)	

**Involved/dissected lymph nodes**				
**Median**	13	15	2	14
**Range**		(4-38)		(6-25)
**≥ 25%**		20		8

**Extracapsular extension**	26	39	10	42

**Stage (Tumor size)**				
**IIA (T1N1)**	13	20	9	37.5
**IIB (T2N1)**	53	80	15	62.5

**Tumor location**				
**Medial**	20	30	6	25
**Central**	16	24	6	25
**Lateral**	30	46	12	50

**Histology**				
**Ductal**	53	80	18	75
**Lobuler**	3	5	2	8
**Other (mixt, mucinous, medullary)**	10	15	4	17

**Histological Grade**				
**I**	10	15	8	33
**II**	39	59	11	41
**III**	17	26	5	26

**Nuclear Grade**				
**I**	14	22	8	33
**II**	26	39	11	46
**III**	26	39	5	21

**Invasion**				
**Lymphatic**	47	71	11	46
**Vascular**	42	64	13	54
**Perineural**	34	52	5	21

**Receptors**				
**Estrogen receptor**	49	74	17	71
**Progesteron receptor**	51	77	17	71

**Systemic therapies**				
**Hormonotherapy**	49	74	17	71
**Chemotherapy**	66	100	23	96

### Treatment

All patients underwent MRM. Median tumor size was 3 cm (range, 1-5). The median number of dissected lymph nodes was 11 (range, 3-37). Following MRM, FAC (5-fluorouracil, adriamycin, cyclophosphamide) or CMF (cyclophosphamide, methotrexate, 5-fluorouracil) adjuvant chemotherapy were administered to 89 of patients (99%) and 66 patients (73%) received adjuvant endocrine therapy for 5 years. One patient only received hormonal therapy. Sixty-six patients (73%) received PMRT (RT group) and 24 patients (27%) did not (no-RT group). All patients were simulated with conventional simulator. The postmastectomy chest wall received a dose of 50 Gy through two tangential fields with 6 MV foton. The mid-axilla received a dose of 50 Gy through an anterior supraclavicular and posterior axillary fields with cobalt-60. Intended dose was given in 25 fractions in a period of 5 week.

### Follow-up

The patients were followed with office visits and physical examinations every 3 months for the first 3 years, every 6 months for the fourth and fifth years, and annually after 5 years. Chest X-ray and liver sonography were requested every 6 months for the first 3 years and annually for the fourth and fifth years. Whole body bone scans were requested annually for the first 5 years. Median follow-up time was 72 months (range, 30-204 months).

### Recurrence

Loco-regional recurrence was identified as local recurrence (chest wall alone) or peripheral lymphatic recurrence (axillary, supraclavicular and internal mammary lymph nodes alone). Local recurrence was defined as any relapse in the area of surgery between the sternum and the anterior axillary line, and below the inferior clavicular fossa and above the seventh rib. Any relapse involving the axillary lymph nodes, and/or other nodes in the infra or supraclavicular fossa or in the internal mammary chain was considered as a regional recurrence. Any recurrence outside these areas was defined as DM.

### Statistical Analysis

LRR and DM rates were calculated by first event analysis. If a distant metastasis developed, then subsequent local failures were censored (vice versa). 5 years actuarial (Kaplain-Meier) DFS and OS rates were computed. When calculating DFS, any failure (local or distant) and/or death from any cause were considered as an event. Statistical significance of outcome differences was determined using the log-rank test. Multivariate analyses of prognostic variables for each outcome were performed using Cox proportional hazards modeling with and without the percentage of positive lymph nodes as a covariate. All statistical tests were 2-tailed, with the level of significance established at p ≤ 0.05. All analyses were conducted using SPSS software (version 16.0.1; SPSS Inc., Chicago, IL).

## Results

The patient characteristics are shown in Table 1. Median lymph node examined in our series was 12 for RT group and 10 for no-RT group. It appears that the two groups were evenly distributed regarding age, menopausal status, dissected lymph nodes, extracapsular extension, histology, high histological grade, hormonal receptor status and systemic treatment. In the contrary involved lymph nodes ratio, greater than 25% was higher for RT group (20% vs 8%). Stage II B (T2N1) patients constituted 80% of the RT group but 62.5% of the no-RT group. The percentages of lymphatic, vascular and perineural invasion, and high nuclear grade, which are known to be poor prognostic factors, were higher in RT group but not statistically significant. Surgical margins were negative for all patients. Tumor location distribution was also different between the groups, medial location being slightly higher in RT group (30% vs 25%). For the entire group, LRR and DM rates were 7% and 20%, respectively, and 5 years actuarial DFS and OS rates were 73.6% and 81.9%, respectively.

**Figure 1:**
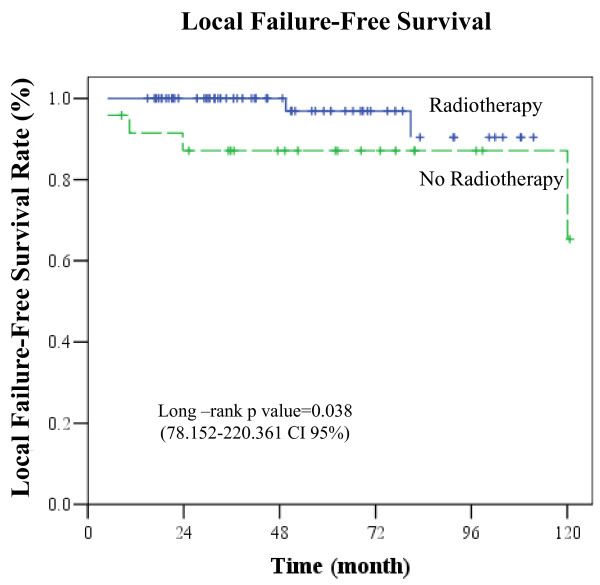
**Kaplan-Meier curve of local failure-free probability (Five year local-regional failure-free survival in RT group 92.6%, no-RT group 87.1%, p = 0.038, (78.152-220.361 CI 95%))**.

**Figure 2:**
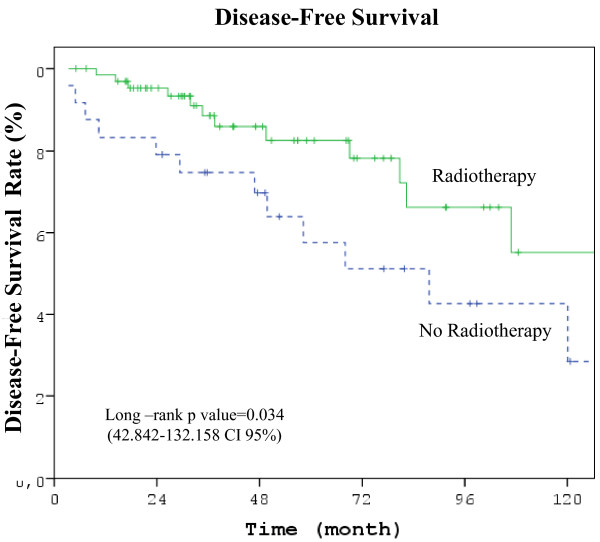
**Kaplan-Meier curve of disease-free probability**. (Five years DFS in RT group 82.4%, no-RT group 52.4%, *p *= 0.034 (42.842-132.158 CI 95%)).

**Figure 3:**
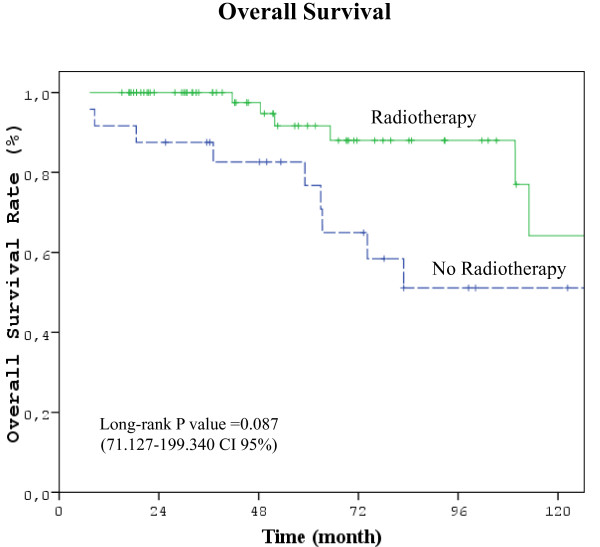
**Kaplan-Meier curve of overall survival probability (Five years OS in RT group 90.2%, no-RT group 61.9%, *p *= 0.087 (71.127-199.340 CI 95%))**.

Differences between RT and no-RT groups were statistically significant for all comparisons in favor of RT group except OS: LRR rate (3%vs 17%, p = 0.038), DM rate (12% vs 42%, p = 0.004), 5 year DFS (82.4% vs 52.4%, p = 0.034, 42.842-132.158 CI 95%), and 5 year OS (90.2% vs 61,9%, p = 0.087, 71.127-199.340 CI95%) (Figure 1, 2, 3) (Table 2).

**Table 2:** The distribution of recurrences and survivals in radiotherapy and no-radiotherapy group

Group	Radiotherapy n = 66		No-Radiotherapy n = 24		P
		
	n	%	n	%	
**Local-regional recurrence**					

Chest wall alone	1	1.5	2	8	

Peripheral lymphatics alone	1	1.5	2	8	

Total LRR^a^	2	3.0	4	17	*0.038*

**Distant metastasis**	8	12	10	42	*0.004*

**Total Events**	10	15	14	58	*0.009*

**Current Status**					

Death	6	9.0	10	42	*0.002*

**DFS**^b ^(5 yrs actuarial)	82.4		52.4		*0.034*

**OS^c ^**(5 yrs actuarial)	90.2		61.9		*0.087*

^a ^Local-regional recurrence, ^b ^Disease-free survival, ^c ^Overall survival

There was no LRR at chest wall and peripheral lymphatics simultaneously nor metachronously. For the entire group, all LRRs occurred as the first event, without DM. Four patients in no-RT group had LRR. All of 8 DM in RT group occurred as the first event without LRR, but only 10 of 14 DM (71%) in no-RT group were the first event, 4 of whom presented with or followed LRR.

Clinical characteristics of patients with LRR are shown in Table 3. LRRs' histologic types were invasive ductal carcinoma for 4 patients and invasive lobular carcinoma for 1 patient and invasive Paget's disease for 1 patient. Remarkably, 3 of the patients with LRR (50%) were younger than 50 of age and premenopousal in no-RT group. Interestingly any patient with LRR had no extranodal extension in our series. As for DM, lymphatic invasion had an impact on LRRs existing in 5 out of 6 (83%). All LRRs in RT group were T2 tumors (3 cm and 4 cm) but only 1 of 4 LRRs in no-RT group was T2 (4.5 cm).

**Table 3:** Clinical characteristics of patients with LRR

Group	RT		No-RT			
**Patient**	**Patient 1**	**Patient 2**	**Patient 1**	**Patient 2**	**Patient 3**	**Patient 4**

**Age**	63	77	43	40	49	55

**Menopouse**	Post	Post	Pre	Post	Pre	Post

**Primary tumor size (cm)**	3	4	2	1.5	2	4.5

**Primary tumor location**	Lat^a^	Lat	Lat	Med^b^	Med	Lat

**Histology**	IDC^c^	IDC	ILC	IDC	IPD^d^	IDC

**Nuclear Grade**	II	I	II	III	II	II

**Histologic Grade**	II	I	II	II	II	I

**Receptor**	-	+	+	+	-	-

**Lymphatic invasion**	+	+	+	+	+	-

**Perineural invasion**	+	-	+	+	-	-

**Extracapsular extension**	-	-	-	-	-	-

**Ratio of lymph node (%)**	11	25	10	25	10	20

**Hormonotherapy**	-	+	+	+	-	-

**Chemotherapy**	+	+	+	+	+	+

**Out-come**	No DM^e^ Alive	DM Death	DM Death	DM Death	DM Death	DM Alive

^a ^Lateral, ^b ^Medial, ^c ^Invasive ductal carcinoma, ^d ^Invasive Paget's disease, ^e ^Distance metastasis

Regarding the ratio of involved nodes, 13 patients in RT group (20%) had a ratio equal to or higher than 25% (highest ratio 37%). Eleven of them (85%) were alive without an event but 2 had recurrences; 1 with LRR and DM simultaneously, the other one with only DM both dying subsequently after treatment. Two patients in no-RT group (8%) had a ratio equal to 25%. One of them developed LRR first followed by DM, the other one had DM without LRR, both dying subsequently after treatment.

There was distant metastatic event in 10 patients in no-RT group (42%). Five of them developed bone metastasis first and liver metastasis subsequently. Two patients had lung metastasis and two had liver metastasis. One patient had mediastinal involvement. On the other hand, 8 patients had DM in RT group (16%). Five of eight patients had bone metastasis only, two had both bone and liver metastases and one had lung metastasis only. Tumor location had also an impact in RT group for DM (eight patients, four of them having their primary tumor location medially and centrally). Most remarkable event of all is that in all patients developing DM but one (in no-RT group) had lymphatic invasion at their primary tumors.

In univaried analysis, lymphatic invasion (*p *= 0.032), perineural invasion (*p *= 0.046), pathological stage (tumor size) (*p *= 0.024), PMRT (*p *= 0.032), LRR (p = 0.047), DM (p = 0.000), and total event (p = 0.002) were prognostic factors which affected DFS. Hormonotherapy could not reach the significance (p = 0.056) for DFS. In multivariate analysis, only DM was independent poor prognostic factor for DFS (p = 0.000).

In univaried analysis, lymphatic invasion (*p *= 0.022), ratio of positive lymph node (*p *= 0.001), PMRT (*p *= 0.087), DM (*p *= 0.000), and total event (*p *= 0.008) were prognostic factors which affected OS. Neither hormonotherapy nor pathological stage could not reach the significance (p = 0.051 and p = 0.065 respectively) for OS. In multivariate analysis, DM (p = 0.000) and lymphatic invasion (p = 0.021) were independent poor prognostic factors for OS. The predictive factors for OS in univariate time-dependent analysis and the independent predictive factors for OS defined by Cox's proportional hazards model are showed in Table 4.

**Table 4:** The impact of clinical and pathologic factors on the overall survival

Factors Assessed	Univariate Analysis			Multivariate Analysis		
	
	Hazard Ratio	95% CI^a^	p^b^	Hazard Ratio	95% CI	p^c^
**Age**						

**≤50 (vs >50)**	0.947	0.32-2.80	0.922			

**Menauposal status**						

**No (vs yes)**	0.966	0.33-2.87	0.951			

**Histologic grade**						

**3 (vs, 1-2)**	1.108	0.36-3.39	1.000			

**Nuclear grade**						

**3 (vs 1-2)**	1.037	0.30-3.62	0.859			

**Lymphatic invasion**						

**Yes (vs absent)**	3.955	0.78-11.3	0.022	3.052	0.98-9.87	0.021

**Vascular invasion**						

**Yes (vs absent)**	1.631	0.46-5.82	0.547			

**Perineural invasion**						

**Yes (vs absent)**	1.771	0.56-5.60	0.409			

**Tumor size**						

**2.1-5 cm (vs ≤2 cm)**	3.323	1.34-12.1	0.013	1.865	1.21-11.8	1.124

**Estrogen receptor**						

**Negative (vs positive)**	1.717	0.44-6.65	0.115			

**Progesteron receptor**						

**Negative (vs positive)**	2.120	0.68-15.35	0.102			

**Ratio of positive lymph node**						

**≥25% (vs <25%)**	3.471	1.14-10.57	0.001	2.139	1.28-11.21	1.239

**Hormonothreapy**						

**No (vs yes)**	2.819	0.91-8.75	0.110			

**Postoperative radiotherapy**						

**No (vs yes)**	2.143	1.22-17.95	0.087	1.253	1.18-16.13	1.124

**Event**						

**Yes (vs absent)**	4.743	1.70-7.28	0.008	2.511	1.74-6.93	0.0812

**Local failure**						

**Yes (vs absent)**	3.462	0.99-30.08	0.067	1.242	1.12-23.7	1.318

**Distant metastasis**						

**Yes (vs absent)**	3.667	1.85-7.26	0.00	5.437	1.92-8.13	0.000

^a ^Confidence interval, ^b ^Kaplan-Meier analysis (log-rank test), ^c ^Cox regression analysis.

None of the patients in the entire group developed brachial plexopathy, any symptomatic pneumonitis and/or severe lymph edema, neither any had secondary cancer in the follow-up period.

## Discussion

Our retrospective study consists of relatively small number of patients and PMRT decision recommended according to the poor prognostic factors of the patient at the discrepancy of the radiation oncologist. Our results showed that PMRT in T1-2 and 1-3 axillary lymph node positive patients caused a statistically significant improvement in the DFS (p = 0.034), in spite of higher risk status in RT group in terms of pathological stage/tumor size, involved lymph node ratio ≥ 25%, high nuclear grade, lymphatic, vascular and perineural invasion. The improvement of OS in RT group was not statistically significant (p = 0.087).

Actually, LRR rates at the long term after MRM may be higher than estimated rates. In our series, LRR rate in no-RT group was higher significantly compared with RT group (17% vs 3%). The trial by Ragaz et al. reports a 5-year LRR rate of 21% among women who did not undergo RT and 10% among those who received RT; 10-year rates were 25% and 13%, respectively [7]. Similarly, Overgaard et al. reports a 114-month rate of LRR alone of 26% for women given chemotherapy without RT and 5% for those given both chemotherapy and RT [3].

During recent decades, as a local treatment RT has been considered to contribute only to local-regional disease control in breast cancer patients. Indeed, data from patients with T1-2 and 1-3 positive nodes supports the contention that PMRT improve not only locoregional control, but also OS [4]. Results of our statistical analysis proved that DM development is an independent prognostic factor for OS. Distant metastasis rate was higher in no-RT group compared to RT group (42% vs 12%), bone and liver metastasis being higher in number as of distribution among metastatic sites. Therefore, the higher LRR rates in no-RT group should be attributed to the fact that locoregional treatment with RT improves survival by reducing LRR which is not prevent significantly by adjuvant chemotherapy and/or hormonotherapy alone [7-10]. All patients in the RT group received systemic adjuvant therapy.

It was state by Truong et al., that the percentage of positive lymph nodes should be consider in adjuvant therapy decisions for women with 1-3 axillary positive lymph nodes who undergo MRM [11]. The presence of 25% or more lymph nodes that are positive identified patients at higher risk of LRR and DM who may benefit from adjuvant RT and more aggressive systemic treatment regimens [11-15]. The median lymph node involvement ratio in our RT and no-RT groups is 15% and 14%, respectively. A great majority of patients in no-RT group had involved lymph nodes of less than 25%, only 2 patients (8%) in this group had this ratio equal to 25%. Despite the situation, their LRR, total event, death rates, and DFS rates were significantly worse than RT group in which 13 patients (20%) had this disadvantage. Another debate about PMRT is about the rational of peripheral lymphatic portal addition to the chest wall irradiation. In our series there were 3 peripheral lymphatic recurrence out of 6 LRR in the entire group, all situated in supraclavicular region suggesting that a small supraclavicular field (excluding humeral head) addition to the chest wall portal would be adequate, which also would prevent a subsequent arm lymphedema.

The question whether with current standards of surgery and systemic adjuvant chemotherapy in this particular subset of intermediate risk patients (N1-3 positive and pT2) with additional risk factors, the prevention of local recurrences through only chest wall irradiation will improve survival, is investigated in an ongoing study of MRC/EORTC SUPREMO trial [6,16].

Arriagada reported a retrospective analysis of IGR database between 1963-1983 on 1105 patients treated by total mastectomy and axillary dissection who did not receive adjuvant chemotherapy or hormonotherapy. The result showed an advantage in favor of PMRT in N1-3 positive patients [17]. In a more recent study Cheng et al reported that in addition to axillary nodal status, estrogen receptor status, lymphovascular space invasion and age at diagnosis were all found to be significant to predict LRR and the impact of PMRT on survival [18]. Beside these individual studies number of reviews and metaanalyses demonstrate an absolute survival benefit of approximately 5% to 10% and approximately 66% to 75% relative reduction in LRR, with PMRT [19-23].

A typical explanation expressed in the following citation from the NIH Consensus Report 2000 [5]: "There is evidence that women with high risk of LRR after MRM benefit from PMRT. This high-risk group includes women with four or more positive nodes or an advanced primary tumor. At this time, the role of PMRT for women with 1 to 3 positive lymph nodes remains uncertain and is being examined in a randomized clinical trial". Many surgeons and radiation oncologist are not recommending PMRT to 1-3 axillary lymph node positive patients with a common understanding that RT will cause to ipsilateral lymphedema of the upper extremity. However, retrospective evaluation of series in the English literature in conjunction with Overgaard et al.'s article opens a new window in the management of 1-3 axillary lymph node positive patients. The authors clearly indicated that PMRT significantly and substantially improved loco-regional control and OS in all node-positive patients. Hence, improvement is as pronounced in patients with 1-3 positive nodes as in patients with 4 or more positive nodes, and nearly the same number of patients is needed to treat to avoid a loco-regional recurrence and/or death in both groups. Therefore, in management of patients with 1-3 axillary lymph nodes positive patients should be reconsidered thoroughly with the guidance of long-term results of studies like DBG 82 and British Columbia randomized trial [2,15].

When making decision for PMRT, radiation oncologist needs additional parameters for this group of patients. As Overgaard et al. mentioned in their article, it is obvious that the number of positive lymph nodes solely is an extremely crude way of defining a potential indication for PMRT. More information may come from other clinicopathologic parameters (e.g., capsule and lymphovascular invasion, malignancy grading, etc.). Moreover, recent years have given increasing knowledge about the prognostic value of new molecular and genetic markers in order to select patients for adjuvant systemic therapy [24-26]. In coming years, these new markers might also be proven as predictors for selecting tumors which are more sensitive to RT than the others.

## Conclusion

Our results share some similar and consistent findings with the recent literature as presented above, in which PMRT resulted to improve local-regional control, DFS and OS. Selection of patients for PMRT in this intermediate risk group is a challenging situation, because some of them have one or more predictor and prognostic factors for failure. It appears that the benefit of RT is worth of the risk of treatment morbidity with accurate selection.

PMRT for T1-2 and N1-3 positive patients has to be reconsidered according to the prognostic factors and the decision has to be made individually with the consideration of long-term morbidity and with the patient approval, until further data are available.

## Competing interests

The authors declare that they have no competing interests.

## Authors' contributions

RC coordinated the entire study. Clinical data collection was done by BD, AO, BU, and KI. Data analysis was done by RC, CU, NT and MS.

The manuscript was prepared by RC and AO. Corrections and/or improvements were suggested by CU, BD, MS, AO, SU, AS, VYC and ZK. Major revisions were done by RC, CU, MS and AO. All authors read and approved the final manuscript.
